# Expression and Functional Role of Sprouty-2 in Breast Morphogenesis

**DOI:** 10.1371/journal.pone.0060798

**Published:** 2013-04-03

**Authors:** Valgardur Sigurdsson, Saevar Ingthorsson, Bylgja Hilmarsdottir, Sigrun M. Gustafsdottir, Sigridur Rut Franzdottir, Ari Jon Arason, Eirikur Steingrimsson, Magnus K. Magnusson, Thorarinn Gudjonsson

**Affiliations:** 1 Stem Cell Research Unit, BioMedical Center, Faculty of Medicine, University of Iceland, Reykjavik, Iceland; 2 Department of Laboratory Hematology, Landspitali University Hospital, Reykjavik, Iceland; 3 Department of Biochemistry and Molecular Biology and BioMedical Center, Faculty of Medicine, University of Iceland, Reykjavik, Iceland; 4 Department of Pharmacology, Faculty of Medicine, University of Iceland, Reykjavik, Iceland; Childrens Hospital Los Angeles, United States of America

## Abstract

Branching morphogenesis is a mechanism used by many species for organogenesis and tissue maintenance. Receptor tyrosine kinases (RTKs), including epidermal growth factor receptor (EGFR) and the sprouty protein family are believed to be critical regulators of branching morphogenesis. The aim of this study was to analyze the expression of Sprouty-2 (SPRY2) in the mammary gland and study its role in branching morphogenesis. Human breast epithelial cells, breast tissue and mouse mammary glands were used for expression studies using immunoblotting, real rime PCR and immunohistochemistry. Knockdown of SPRY2 in the breast epithelial stem cell line D492 was done by lentiviral transduction of shRNA constructs targeting *SPRY2*. Three dimensional culture of D492 with or without endothelial cells was done in reconstituted basement membrane matrix. We show that in the human breast, SPRY2 is predominantly expressed in the luminal epithelial cells of both ducts and lobuli. In the mouse mammary gland, SPRY2 expression is low or absent in the virgin state, while in the pregnant mammary gland SPRY2 is expressed at branching epithelial buds with increased expression during lactation. This expression pattern is closely associated with the activation of the EGFR pathway. Using D492 which generates branching structures in three-dimensional (3D) culture, we show that SPRY2 expression is low during initiation of branching with subsequent increase throughout the branching process. Immunostaining locates expression of phosphorylated SPRY2 and EGFR at the tip of lobular-like, branching ends. SPRY2 knockdown (KD) resulted in increased migration, increased pERK and larger and more complex branching structures indicating a loss of negative feedback control during branching morphogenesis. In D492 co-cultures with endothelial cells, D492 SPRY2 KD generates spindle-like colonies that bear hallmarks of epithelial to mesenchymal transition. These data indicate that SPRY2 is an important regulator of branching morphogenesis and epithelial to mesenchymal transition in the mammary gland.

## Introduction

Branching morphogenesis is a highly conserved developmental process, where epithelial-based organs are able to increase their surface area and form the correct functional histoarchitecture [1], [2]. This process gives rise to the airways of the lungs [3], the urine collecting ducts [4], the prostate [5], salivary glands [6] and the mammary glands [7], [8].

The molecular events that induce and regulate branching morphogenesis are highly conserved between different organs and between different species [9]. Receptor tyrosine kinases (RTKs), such as fibroblast growth factor receptors (FGFRs) and epidermal growth factor receptors (EGFRs) are key mediators of signals that regulate proliferation, differentiation and branching morphogenesis in the mammary gland [1], [10]. Extracellular cues such as FGFs and EGFs act via their respective receptors to activate intracellular pathways, such as the mitogen-activated protein kinase (MAPK) and phosphatidylinositol 3-kinase (PI-3 kinase) pathways which are critical for proper development of branched organs [1]. In addition, aberrant expression and activation of RTKs such as the EGFR family is common in a number of cancers including breast cancer (reviewed in [11]). Molecular signaling in branching morphogenesis must be precisely regulated both spatially and temporally to ensure normal homeostasis. Recent studies have underscored the importance of negative feedback control of RTK signaling for ensuring correct cell fate and morphogenesis [12]. Sprouty, initially shown to be critical for tracheal development in Drosophila [13], is now known to act as a conserved negative feedback regulator of RTK signaling in higher eukaryotes [14]–[20]. There are four mammalian Sprouty proteins (SPRY1–4) and they have been proposed to participate in a classical negative feedback loop of RTK signaling through the MAKP pathway [21]. However, detailed molecular mechanisms of action of the sprouty proteins have not been fully elucidated. The studies of sprouty in the mammals have thus far mostly focused on the regulation of FGFR and EGFR [21]. Sprouty proteins have been identified as regulators of FGFR, c-Met and EGFR signaling in lung, kidney and vasculogenesis but their role in the human breast gland morphogenesis has not been systematically analyzed [22]. Although, sprouty proteins are considered negative inhibitors of RTK signaling their role in maintaining signal activity has been reported. Thus, SPRY2 has been shown to delay EGFR breakdown in endosomes after internalization by binding the catalytic RING Finger of Casitas B-lineage lymphoma (c-Cbl), an E3 ubiquitin ligase that has been identified to target EGFR degradation. SPRY2 sequester c-Cbl molecules from activated EGFR and disregulate EGFR ubiquitination and downregulation, thereby potentiating the amplitude and longevity of intracellular signals [23], [24].

In mouse mammary glands the branching ducts are embedded in fat-rich stroma whereas in humans, breast ducts are more elaborate and terminate in the lobuli commonly referred to as the terminal duct lobular units (TDLU) [25]. The TDLUs are composed of differentiated luminal- (LEP) and surrounding myoepithelial (MEP) cells, separated from the stroma by a basement membrane. Branching morphogenesis in the mammary gland is believed to occur through collective migration of both LEP and MEP cells where epithelial cells at the branching end lose adhesion and acquire transient epithelial to mesenchymal transition (EMT) resulting in increased motility [7], [26]. Temporal EMT phenotypes have also been linked to cancer progression and metastasis [26]–[28]. This temporal activation of EMT in both cancer progression and branching morphogenesis highlights the importance of understanding the molecular regulators of breast morphogenesis. Indeed, disruption in the regulation of RTKs, critical regulators of branching morphogenesis, is also a major factor seen in many cancer forms, including breast cancers [1]. Lo et al. [29] have shown that SPRY2 expression is suppressed in breast cancers suggesting that SPRY2 might function as a tumor suppressor. Interestingly, Faratian et al. [30] have recently shown that reduced expression of SPRY2 is an independent prognostic factor in HER2 positive breast cancer. These data link candidate morphogenic pathways to breast cancer progression.

Three-dimensional cultures have proven to be important tools for recapitulating an *in vivo* like context in the mammary gland [31], [32]. We have previously shown that D492, an epithelial cell line with stem cell properties, generates TDLU-like structures in 3D culture [33], [34]. D492 is thus a good model to dissect molecular mechanisms regulating branching morphogenesis. We have also shown that endothelial cells stimulate growth and morphogenesis of breast and lung epithelial cells [35], [36]. Most recently, we demonstrated that endothelial cells facilitate branching morphogenesis of D492 in co-culture and furthermore induces a subpopulation of D492 to generate spindle-like colonies through an EMT conversion [37]. Here, we show that SPRY2 is predominantly expressed in luminal epithelial cells of duct and lobuli in human breast tissue. We also show that SPRY2 is highly expressed in the pregnant and lactating mouse mammary gland with phosphorylated SPRY2 peaking during pregnancy. Expression of SPRY2 is associated with expression of phosphorylated EGFR (pY1068) and activation of the downstream MAPK signaling pathway. Using D492, we show that SPRY2 is expressed at the branching tips and suppression of SPRY2 through shRNA gene knockdown increases branching morphogenesis and promotes epithelial to mesenchymal transition when cultured with endothelial cells.

## Materials and Methods

### Cell culture

The breast epithelial stem cell line D492 was maintained in H14 medium [38], consisting of DMEM/F12, 50 IU/ml penicillin, 50 µg/ml streptomycin (Invitrogen), 250 ng/ml insulin, 10 µg/ml transferrin, 2.6 ng/ml sodium selenite, 0.1 nM estradiol, 0.5 µg/ml hydrocortisone, 5 µg/ml prolactin (SIGMA) and 10 ng/ml EGF (Peprotech). Primary LEPs and MEPs were maintained on CDM3 and CDM4 as previously described [35], [39]. Primary human BRENCs were isolated from breast reduction mammoplasties as previously described [40] and cultured on endothelial growth medium (EGM-2) (Lonza) +5% FBS (Invitrogen).

### Preparation of 3D mono- and co-cultures

3D monocultures were carried out in 96 well culture plates (Becton Dickinson, BD, Falcon). 7×10^3^, 1×10^4^ and 1.3×10^4^ D492 cells were suspended in 300 µl of reconstituted basement membrane (rBM) purchased as matrigel (BD). Co-culture experiments were carried out with 1×10^3^ D492 mixed with 5×10^4^ BRENCs. 100 µl of mixed cells / rBM were seeded in each well in a 96 well plate and cultured on H14 (Monoculture) or EGM5 (Co-culture) for 16 days.

### Isolation and processing of mammary glands and 3D cell cultures

Human tissue from breast reductions was used for immunohistochemistry and for isolation of primary breast epithelial cells. Primary LEPs and MEPs were isolated by magnetic cell sorting (MACS) as previously described [39]. Murine mammary glands were dissected from C57BL/6 mice at the following stages: 6 week old virgins, day 15 of pregnancy and day 2 of lactation. Mammary glands were snap frozen in liquid nitrogen and preserved at –80°C. Isolation of colonies from 3D cell culture was done as previously described by gentle dissociation in PBS-EDTA buffer [41].

### Immunochemistry

Formalin-fixed, paraffin embedded human tissue blocks from reduction mammoplasty biopsies were cut into 5 µm serial sections and mounted on slides. Sections were deparaffinized and rehydrated in xylene and ethanol. Antigen retrieval was done by boiling in EDTA buffer for 15 minutes. Frozen mouse mammary glands were cryosectioned at 15 µm setting following formalin fixation. The following primary antibodies were used; Sprouty-2 (#07-524, Upstate/Millipore), CD-31 (M0823, DakoCytomation), Keratin 19 (ab7754, Abcam), Keratin 14 (NCL-LL002, NovoCastra), PCNA (ab29, Abcam), EGFR (#4267, Cell Signaling), p-EGFR (Tyr1068) (#3777, Cell Signaling), ki67 (Abcam, ab833), E-Cadherin (BD Biosciences, cat. 610182), N-Cadherin (BD Biosciences, cat. 610921).Fluorescent nuclear counterstain, TO-PRO-3 (Invitrogen) was used in immunofluorescence. Specimens were visualized on a Zeiss LSM 5 Pascal laser-scanning microscope (Carl Zeiss).

### In situ Proximity Ligation assay

Protein phosphorylation of Spry2 was studied *in situ* by Proximity Ligation assay (PLA) using the Duolink(R) kit (Olink Bioscience, Uppsala, Sweden) [42]. Sections from mouse mammary glands and 3D cultures were fixed with PFA, blocked and incubated with primary antibodies, Sprouty-2 at 1∶50 dilution (#07-524, Upstate/Millipore), and P-Tyr-100 at 1∶100 dilution (#9411S, Cell Signaling), overnight at 4°C. The remaining steps of the PLA were performed as suggested by the kit manufacturer. Cells were incubated with secondary anti-mouse PLUS and anti-rabbit MINUS probes. Pairwise binding to the target allowed free oligonucleotide ends of the probes to come into close proximity, and the free ends enabled formation of circular DNA molecules through ligation. The DNA circles were then amplified and detected by hybridization of fluorescently labeled oligonucleotides. Nuclei were counterstained with TO-PRO 3 (Invitrogen). Single phosphorylated Spry2 molecules were visualized using LSM 5 Pascal confocal microscope (Carl Zeiss, Jena).

### In Situ Hybridization

2981bp segment of Spry2 DNA was amplified from human blood cells, with forward (CTAAGCCTGCTGGAGTGACC) and reverse (GGAACTTTGAAAAACCAACA) primers generated with online Biology Workbench (http://workbench.sdsc.edu). DIG-labeled RNA probe synthesis was performed according to the manufactureŕs instructions (DIG RNA Labeling Mix, Roche). Paraffin embedded normal breast tissue slides were processed and then treated for 10 minutes with proteinase K (Fermentas), and with acetic anhydride / triethanolamine (Sigma) for 10 minutes. Before hybridization, the samples were prepared in pre-hybridization buffer for 2 hours. Hybridization was carried out for 12–16 hours. Slides were then washed and incubated with Anti-Dioxigenin-AP fab fragments (Roche) antibody for 4 hours at RT. Color development was carried out with NBT/BCIP buffer (Sigma) for 4 hours at RT in dark.

### Western blotting

Equal amounts (5 µg) of proteins were separated on 10% NuPage Bis-Tris gels (Invitrogen) and transferred to a PVDF membrane (Invitrogen). Antibodies: Sprouty-2 (1∶2000, #07-524, Upstate/Millipore), pERK (1∶2000, #4695, Cell Signaling), pERK (1∶2000, #9101, Cell Signaling), and β-actin (1∶5000; ab3280, Abcam). Membranes were visualized with ECL+ after incubation with anti-mouse or rabbit secondary antibodies (1∶5000) (GE healthcare).

### Q-RT-PCR

Total RNA was extracted with Trizol (Invitrogen), DNAase treated and reverse transcribed with Hexanucleotides using ReverAid (#K1622, Fermentas). Resulting cDNA was used for Q-RT-PCR, in master mix (Applied Biosystems) with primer pairs and probes for Spry2 (Hs00183386_m1, AB) and GAPDH (Applied Biosystems). Experiments were done in triplicate on 7500 Real Time PCR System (Applied Biosystems). SPRY2 mRNA levels were normalized to GAPD and relative mRNA differences was calculated with the 2^ΔCt^ Method.

### shRNA knockdown of SPRY2

Three separate pGIPZ lentiviral shRNA constructs targeting SPRY2 transcripts were purchased from Open Biosystems (RHS4430-101098640 (KD3), RHS4430-101103852 (KD2), RMM1766-96881511 (KD1)). A non-silencing construct (RHS4346) was used as a control. All constructs contained both a puromycin selection marker and green fluorescent protein (GFP). Viral particles were produced in HEK-293T cells using Arrest-In transfection reagent (ATR1740; Open Biosystems) according to instructions. Virus-containing supernatants were collected at 48 hours after transfection and target cells were infected in the presence of 8 µg/µl polybrene. Stable, D492^SPRY2 KD^ cells were established by puromycin selection (3 µg/µl) followed by flow-sorting, selecting GFP expressing cells.

### Migration and proliferation assay

For migration experiments, a total of 2.5×10^4^ starved cells were seeded in DMEM/F12 basic medium on collagen coated upper compartment of a transwell Boyden chamber (Corning) with an 8 µm pore size. EGM5 medium was used as a chemoattractant in the lower chamber. After 18h incubation, cells in the upper chamber were removed with a cotton swab and migrated cells on the bottom surface stained with 0.1% crystal violet. Cells were counted in three representative fields in each filter. In the proliferation assay, 10^4^ cells were seeded per well in a 24 well plate (Falcon, BD). Cells were fixed with formalin and stained with 0.1% crystal violet (days 1–5), washed and left to dry. The crystal violet staining in each well was dissolved in 10% acetic acid and measured at 570 nm in a plate reader.

### Statistical analysis

All migration and 3D culture experiments were performed in triplicate. Data is presented as mean +SEM from number of independent experiments as indicated. Statistical analysis was performed by two-tailed Students T-test using GraphPad. P values of <0.05 were considered to be statistically significant.

### Ethics Statement

The breast tissue samples were provided by written informed consent from women undergoing reduction mammoplasty. This procedure has been approved by the National Bioethics Committee of Iceland, Reference number VSNa2001050056. The Committee on the use of Experimental Animals (Tilraunadýranefnd) approves all protocols for experiments on animals performed in Iceland. The committee does not require special approval for collecting tissues after euthanasia, as was done in this report. Details of animal welfare; Mice were housed in Micro Isolator cages (Lab Products Inc.) according to the guidelines set out in the recommendation of the EU commission (2007/526/EC - June 18, 2007) for accommodation and care of animals used for experimental and other scientific purposes, and according to Icelandic law (number 15/1994) and regulations (number 279/2002). Mice were euthanized by inhalation of high concentrations of CO2. This method is classified as “acceptable” in the recommendations of the Panel on Euthanasia of the American Veterinary Medical Association.

## Results

### SPRY2 is predominantly expressed in luminal epithelial cells in the human breast gland

**Figure 1:**
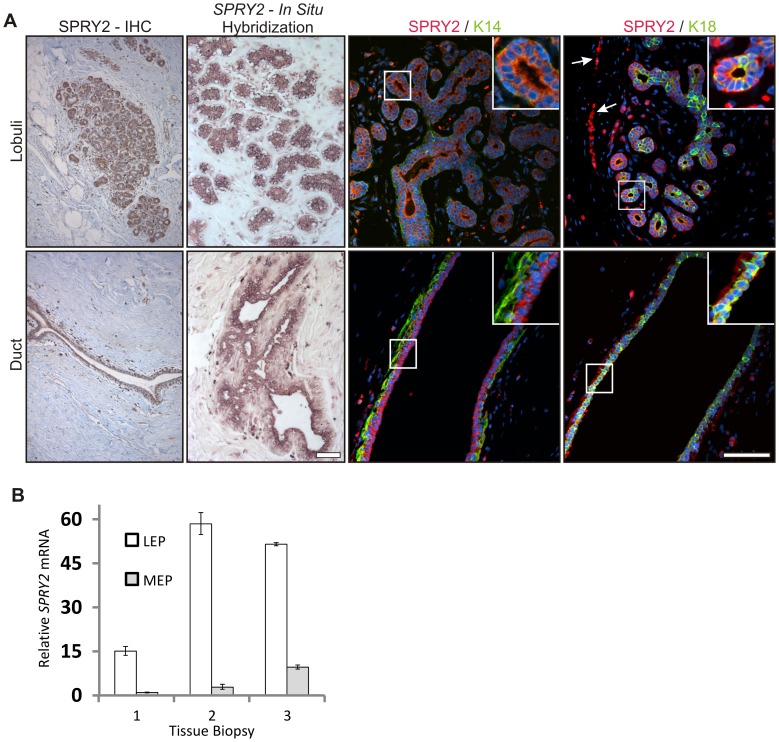
Expression of SPRY2 in lobules and ducts in the normal human breast gland. Expression of SPRY2 was evaluated in normal human breast tissue derived from reduction mammoplasty biopsies. *A) Expression of SPRY2 is most prominent in the luminal epithelial cells.* SPRY2 expression was predominantly found within the epithelial compartment of duct and lobuli as evidenced by immunohistochemistry and in situ hybridization. SPRY2 was predominantly expressed in luminal epithelial cells both in ducts and lobuli. SPRY2 was co-stained for K14 (myoepithelial cells) and K18 (luminal epithelial cells). Note the co-expression of SPRY2 and K18 in luminal epithelial cells. SPRY2 expression was also presence in the stroma, most likely in endothelial cells (arrows). Sections were counterstained with TOPRO-3. Bar  = 100 µm. *B) Expression differences of SPRY2 in luminal- and myoepithelial cells.* Real time PCR was used to quantify expression difference of *SPRY2* between luminal- and myoepithelial cells. *SPRY2* expression was generally low in myoepithelial cells compared to luminal epithelial cells that expressed up to 58 fold more *SPRY2*. Measurement was done in paired luminal and myoepithelial cells from three different biopsies.

To explore the expression of SPRY2 in the human breast gland we performed immunostaining and *in situ* hybridization against SPRY2 in tissues representing the adult non-pregnant human mammary gland from reduction mammoplasties. SPRY2 expression was seen in epithelial cells, both in large ducts and in the terminal duct lobular units (TDLU) (Fig. 1A). Dual labeling with antibodies against SPRY2 and the linage restricted markers cytokeratin (CK) 18 (luminal epithelial cells) or CK14 (myoepithelial cell), demonstrated that SPRY2 was predominantly expressed within the luminal epithelial compartment (Fig. 1A). SPRY2 was also detected in discreet areas in the stroma, most likely endothelial cells (Fig. 1A arrows). This was supported by analyzing the expression of SPRY2 in purified myoepithelial and luminal epithelial cells isolated from three different breast tissue samples using quantitative real-time PCR. Luminal epithelial cells showed 15–58 fold higher expression of SPRY2 compared to myoepithelial cells (Fig. 1B) in cell purifications from three different breast tissue samples.

### SPRY2 expression is associated with activated EGFR signaling in the pregnant and lactating mouse mammary gland

**Figure 2:**
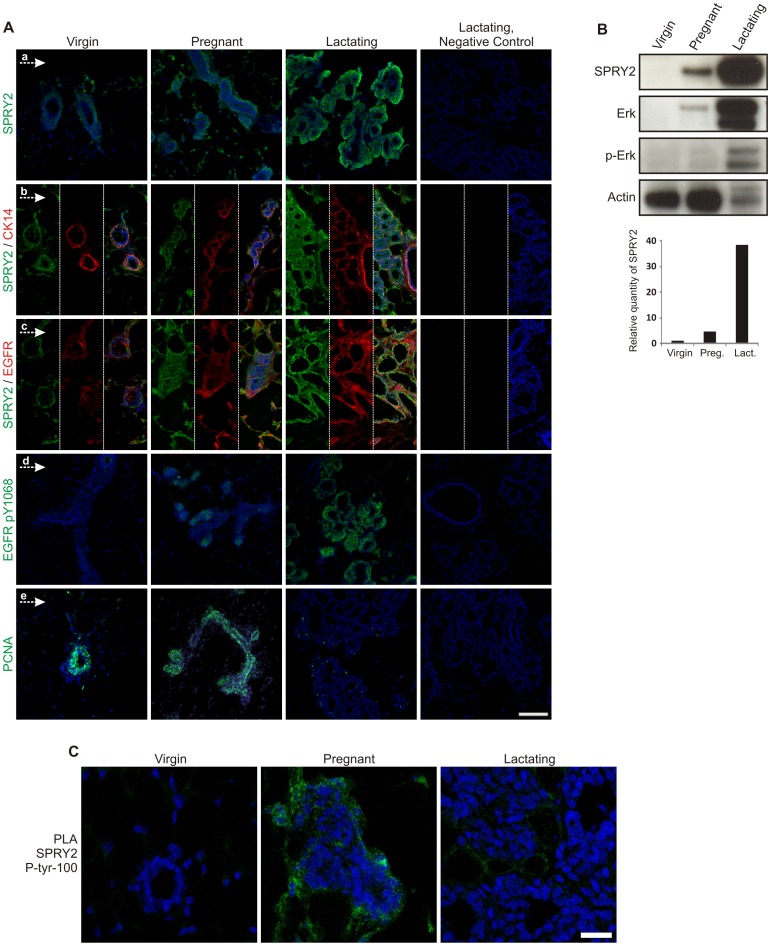
Expression of SPRY2 in virgin, pregnant and lactating mouse mammary gland. *A) Expression of SPRY2 and pEGFR is inversely correlated with cell proliferation.* Low expression of SPRY2 is found within the virgin gland with few positive stromal cells (a). Note, increased stromal expression of SPRY2 in pregnant gland accompanied with expression in myoepithelial cell as evidenced by double staining of SPRY2 and the myoepithelial marker CK14 (b). Dramatic increase in SPRY2 expression is seen during lactation (a and b). SPRY2 and EGFR show similar expression pattern at all stages (c) with pEGFR expression seen at terminal buds in pregnant gland. Dramatic increase in pEGFR expression is seen in the lactating gland. Similar expression is found for SPRY2 and pEGFR in lactating gland. Proliferation is increased from virgin to pregnant gland but is reduced during lactation, with only few PCNA positive cells left. Cells counterstained with TOPRO-3, Bar = 100 µm. *B) SPRY2 expression is highest during lactation accompanied by activation of Erk/MAPK pathway.* Western blot demonstrated the expression differences of SPRY2 in virgin, pregnant and lactating glands. There is over 38 fold increase in SPRY2 expression during lactation compared to virgin state. Total ERK and pERK is also significantly increased during lactation. Actin was used as a loading control. *C) SPRY2 activity is peaking during pregnancy in the mouse mammary gland.* Using proximity ligation assay it was shown that phosphorylated SPRY2 was significantly more expressed during pregnancy compared to virgin and lactating gland. Bar = 25 µm.

A disadvantage of studying sprouty expression in tissue from reduction mammoplasty is that we are unable to analyze the temporal expression changes during different stages of branching morphogenesis. Therefore, we analyzed the expression of SPRY2 in mouse mammary gland at different stages of development. We isolated mammary glands from virgin, pregnant and lactating mice and analyzed total SPRY2 expression and pSPRY2 and correlated this with pEGFR level. Low level of SPRY2 was seen in the virgin gland. Expression was more prominent in the pregnant gland where the expression was co-localized with myoepithelial cells as evidenced by co-staining for SPRY2 and CK14 (Fig. 2Aa and b). SPRY2 expression reached its highest levels during lactation. Total EGFR showed similar expression pattern as SPRY2. pEGFR was low or absent in the virgin mammary gland but increased focally at branching end buds in the pregnant gland. Furthermore, a dramatic increase in pEGFR was seen in end buds during lactation (Fig. 2Ad). (Fig. 2A). The high EGFR phosphorylation and SPRY2 expression in the lactating state was not associated with cell proliferation marker PCNA (Fig. 2Ae). In order to get quantitative information on SPRY2 expression, western blot analysis was performed on mouse mammary gland tissues. SPRY2 was low or not detected in the virgin mammary gland whereas it was expressed at low levels in pregnant and high levels in lactating mammary glands (Fig. 2B and Figure S1). ERK1/2 mediates signaling through the RAS/MAPK cascade downstream of EGFR. Total ERK expression was correlated with total SPRY2 expression with highest levels seen during lactation. In contrast, phosphorylated ERK1/2 was increased substantially in lactating state only (Fig. 2B). There is no commercially available antibody against phosphorylated SPRY2. To analyze the tyrosine phosphorylation status of SPRY2 in the virgin, pregnant and lactating gland we carried out proximity ligation assay (PLA) using antibodies against SPRY2 and phosphorylated tyrosine residues (see material and methods for details). The PLA assay demonstrated strong SPRY2 phosphorylation in mammary glands from pregnant mice compared to virgin or lactating mice (Fig. 2C). These data suggest that loss of pSPRY2 during lactation is accompanied by increased ERK activity.

### Spatial and temporal expression of SPRY2 and pEGFR during branching morphogenesis of breast epithelial cells in 3D culture

**Figure 3:**
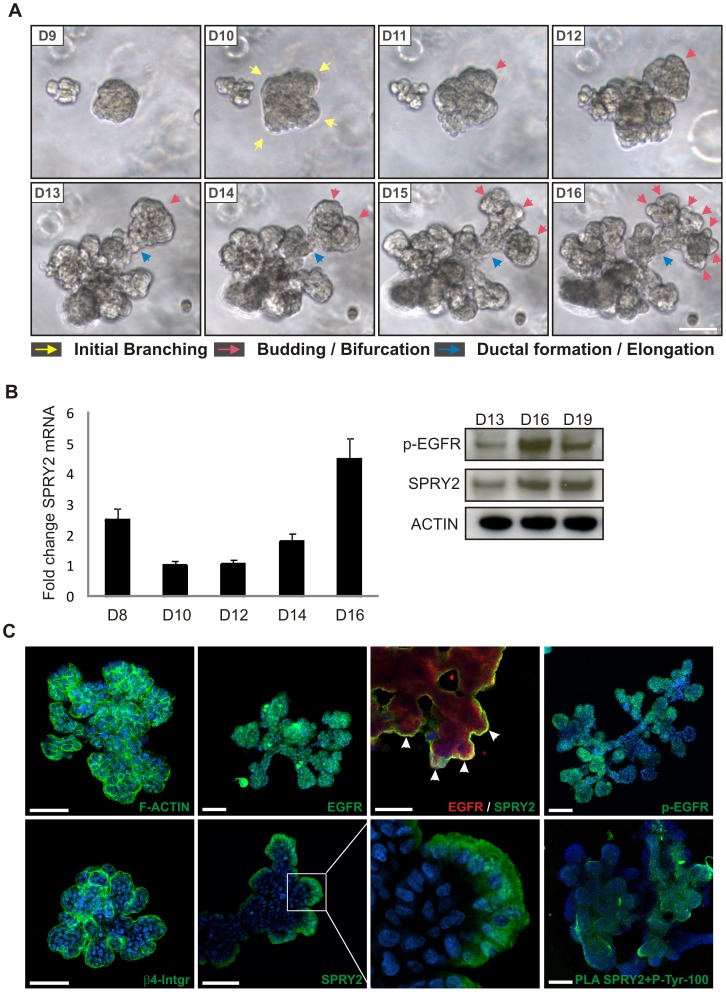
SPRY2 expression is correlated with critical points in branching morphogenesis of D492 breast stem cell line. *A) D492 cells generate branching structures when cultured in rBM.* When seeded in rBM D492 cells generate TDLU-like structures. By generating in vitro TDLU-like structures it is possible to follow individual steps in the branching morphogenesis process. Until day 8 or 9 cells grow as single colonies. First sign of initial budding occurs at day 10 and 11 (yellow arrows) followed by duct elongation and bifurcation (blue and red arrows), respectively. *B) SPRY2 expression shows a dramatic shift during TDLU formation in 3D culture.* Colonies were isolated from 3D cultures at different time points as indicated. Initially at day 8 there is relative high expression of SPRY2 mRNA but its expression is reduced during initial budding but increases again during duct elongation and further bifurcation of complex branching. Western blot confirms that SPRY2 levels increase up to day 16 and remain high while pEGFR is slightly decreasing for day 16 to day 19. Actin was used as a loading control. *C) pEGFR and SPRY2 are expressed at the growing tips of TDLU-like structures.* D492-derived TDLU-like structures generated in 3D culture were stained with antibodies against SPRY2, EGFR, pEGFR, β4-integrin and F-actin. pEGFR was predominantly expressed at the branching tips while total EGFR had a more general distribution. SPRY2 was also expressed at branching tips but not in clefts. Co-staining of SPRY2 and EGFR show strong expression at the branching tips (arrows). F-actin staining gives a general outlook of a branching colony while β4-integrin outlines their connection to the surrounding rBM matrix. Phosphorylated SPRY2 (right) was analyzed using proximity ligation assay as described above. pSPRY2 was predominantly expressed at the branching tips showing similar pattern as total SPRY2. Cells were counterstained with TOPRO-3 nuclear stain. Bar = 100 µm.

To directly study the functional role of SPRY2 in branching morphogenesis of the human breast epithelium we used the D492 cell line cultured in 3D reconstituted basement membrane (rBM). D492 has stem cell properties, i.e. it can differentiate into luminal- and myoepithelial cells and forms TDLU-like colonies through branching morphogenesis when cultured within a 3D rBM [33], [34]. We first analyzed temporal expression of SPRY2 during TDLU formation in 3D rBM. D492 cells undergo most extensive branching during days 10–16 in 3D rBM culture (Fig. 3A). Initially, D492 forms solid round colonies that start to branch on days 10–12 (initial branching). After the first branching event ductal structures elongate and secondary branching occurs with bifurcation at the lobular-like ends (Fig. 3A). To analyze SPRY2 expression we isolated mRNA from culture days, 8, 10, 12, 14 and 16. Pre-branching (day 8), round colonies show high expression of SPRY2. Interestingly, during the initial branching period (days 10–12) the expression of SPRY2 decreases. At day 16 elaborate TDLU-like structures have formed and the expression of SPRY2 increases to more than 4-fold levels compared to day 10 (Fig. 3B). Expression was also confirmed with an immunoblot on D13, D16 and D19, showing the increase in SPRY2 expression from D13 to D16. pEGFR expression pattern was similar with increasing levels from D13 to D16 while decreasing on D19 when branching has stopped (Fig. 3B). This expression pattern suggests that SPRY2 might have a regulatory role during the temporal formation of branching structures and the formation of lobular units at the ductal ends. In support of this, immunofluorescent staining of branching colonies at day 16 shows that SPRY2 expression is mainly concentrated at the branching, lobular-like tips but is lowered at sites of cleft formation (Fig. 3C). The location of SPRY2 at day 16 is similar to that of pEGFR at branching tips while staining for total EGFR has a more general distribution in the branching colonies (Fig. 3C). Co-staining of EGFR and SPRY2 demonstrate co-localization at the edge of the branching structures. Phosphorylated SPRY2 followed the same pattern as pEGFR and total SPRY2 as seen using the PLA (3C). Staining for ß4-integrin and F-actin expression show the general outlines of the branching structures and its connection to the surrounding basement membrane (Fig. 3C).

### SPRY2 knockdown in D492 stimulates branching morphogenesis

**Figure 4:**
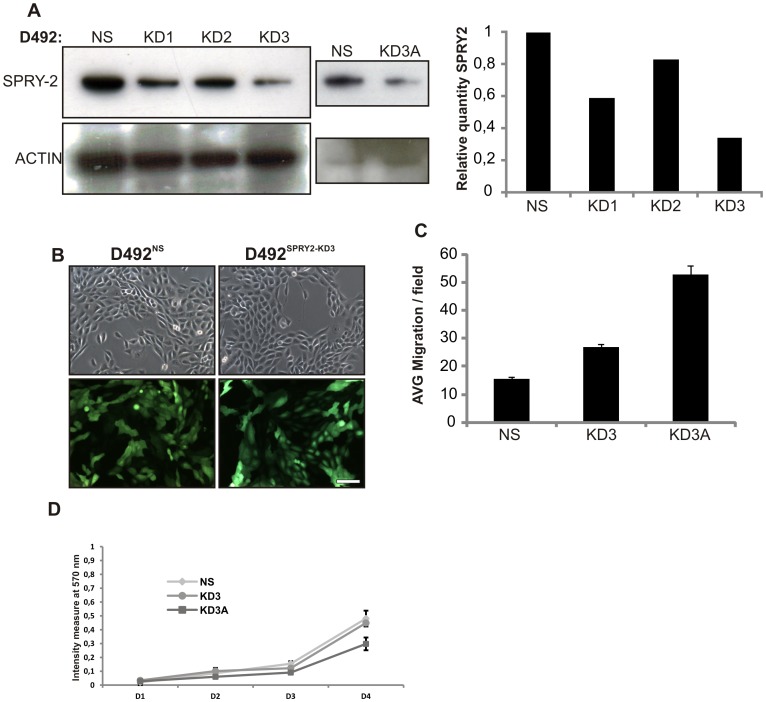
SPRY2 Knockdown in D492 breast epithelial stem cell line. *A) D492 cells show significant knockdown of SPRY2.* D492 were transfected with non-silencing (NS) shRNA and different version of knockdown (KD) shRNA against SPRY2. KD3 showed most efficient knockdown (70%) measured by western blot. KD3A is a single cell cloned subline from KD3. *B) D492SPRY2^-KD3^ retains an epithelial phenotype in monolayer culture.* No phenotypic differences were observed in monolayer of D492^NS^ and *D492^SPRY2-KD3^* (upper row). Transfection efficacy was evaluated by GFP (lower row). *C) D492^SPRY2-KD^ cells have acquired increased migration potential.* When plated on porous transwell filter *D492^SPRY2-KD3^* showed increased migration compared to D492^NS^. Single cell derived clone KD3A from KD3 had the highest migration potential. *D) SPRY2 knockdown has no effect on cell proliferation*. Monolayer proliferation of D492^NS^, D492^SPRY2-KD3^ and D492^SPR2-KD3A^ was evaluated at different time points, as indicated. There was no remarkable difference in the proliferation rate of the NS and KD cells, although at day 4 D492^SPRY2-KD3A^ seemed to proliferate slightly less.

**Figure 5:**
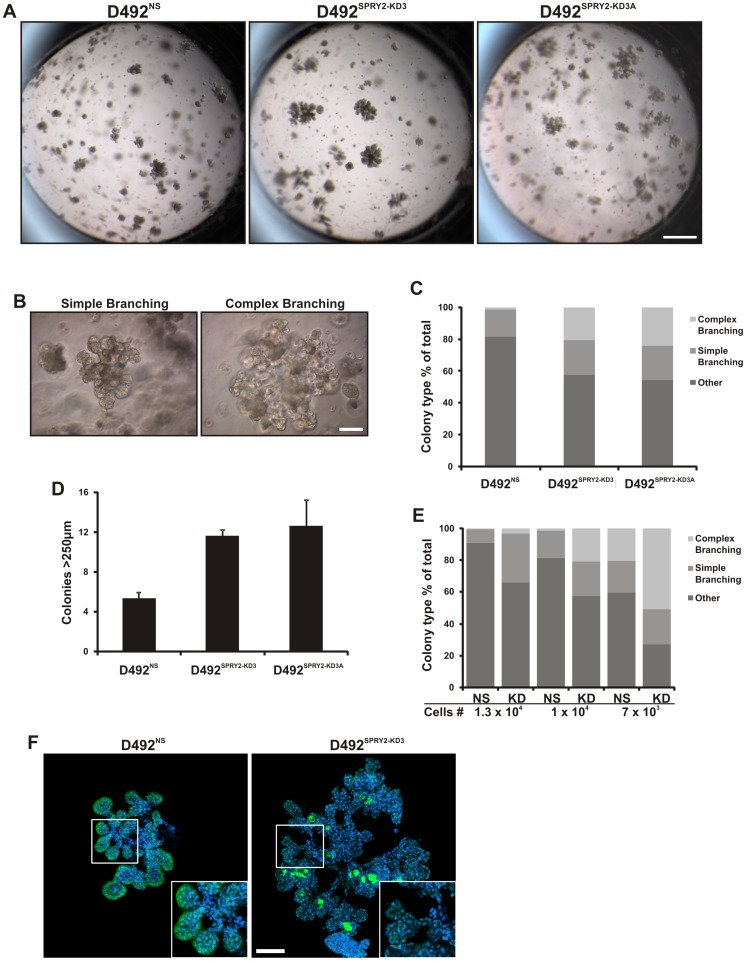
SPRY2 Knockdown in D492 promotes increased branching morphogenesis. *A) SPRY2-KD resulted in increased branching colonies in 3D culture.* Stereoscopic images of representative 3D rBM gels for D492^NS^, D492^SPRY22-KD3^ and D492^SPRY2-KD3A^ showing increased branching upon SPR2 knockdown. Bar  =  2mm. *B) Types of morphogenesis in 3D culture*. Epithelial colonies were divided into three morphotypes: simple branching, complex branching and other (mostly solid round). Representative images of simple- and complex branching are shown. *C) 3D morphogenesis of D492^NS,^ D492^SPRY2-KD3^ and D492^SPRY2-KD3A^ cells.* In a setup with 10^4^ cells both D492^SPRY2-KD3^ and D492^SPRY2-KD3A^ cells showed an increase in simple- and complex branching pattern. Bar  = 100 µm. *D) Large complex colonies in 3D rBM culture.* Complex branching colonies over 250 µm were counted. This showed a 2 fold increase in size of the SPRY2 KD cells. *E) 3D morphogenesis of D492^NS,^ D492^SPRY2-KD3^ and D492^SPRY2-KD3A^ cells with variable amount of cells.* In a setup using 1.3×10^4^, 10^4^ and 7×10^3^ cells, the SPR2-KD cells showed superior branching abilities compared to NS cells. *F) SPRY2 expression in branching colonies from D492^NS^ and D492^SPRY2-KD3^*. As seen before SPRY2 was located at branching tips in NS cells. SPRY2 KD cells showed reduced expression of SPRY2 with some areas of diffuse staining. Bar  = 100 µm.

To further explore the functional role of SPRY2 in the regulation of branching morphogenesis we knocked down SPRY2 in D492 cells and explored their proliferative, migratory and morphogenic potential. We used a lentiviral based shRNA knockdown where D492 were transduced with a GFP-containing non-silencing (NS) control and 3 different knockdown (KD) shRNA constructs (SPRY2-KD1, SPRY2-KD2 and SPRY2-KD3) targeting SPRY2. The SPRY2-KD3 construct was most effective, decreasing SPRY2 expression levels 4 fold (Fig. 4A). Thus, we used this knockdown cell line and a single cell subclone referred to as SPRY2-KD3A. No morphological difference was seen between NS cells and KD3A cells when visualized in a monolayer (Fig. 4B) but D492^SPRY2-KD3^ and D492^SPRY2-KD3A^ showed increased migration compared to D492^NS^ cells (Fig. 4C). There was no significant difference in the proliferation of D492^SPRY2-KD3^ and D492^SPRY2-NS^ cells (Fig. 4D). However, increased expression of pERK is seen in D492^SPRY2-KD3A^ (Figure S2) which could explain the migration ability of these cells. To analyze the effects of SPRY2 knockdown on branching morphogenesis we compared D492^SPRY2-NS^, D492^SPRY2-KD3^ and D492^SPRY2-KD3A^ in 3D rBM culture. D492^SPRY2-NS^ generated *in vivo*-like 3D branching colonies similar to wild type D492 while D492^SPRY2-KD3^ and D492^SPRY2-KD3A^ showed increased branching (Fig. 5A). The effect of SPRY2 knockdown was quantified by counting colonies of simple/early branching, complex/late branching and other (solid round colonies) morphology (Fig. 5B). In a setup with 1×10^4^ cells both D492^SPRY2-KD3^ and D492^SPRY2-KD3A^ formed more branching colonies in total and substantially more colonies that showed complex branching phenotype compared to D492^SPRY2-NS^ cells (Fig. 5C). Large complex colonies (>250 µm) were twice as common in both D492^SPRY2KD3^ and D492^SPRY2-KD3A^ compared to D492^SPRY2-NS^ cells (Fig. 5D). All cell lines were cultured in three different cell concentrations (1.3×10^4^, 1×10^4^ and 7×10^3^) due to the fact that different degree of branching is observed with different number of cells seeded in the rBM. In general less branching was seen in cultures with higher cell density but the D492^SPRY2-KD3^ cells formed more branching colonies in all cell concentrations (Fig. 5E). When we looked at the expression of SPRY2 at D16 in D492^SPRY2-NS^ and D492^SPRY2-KD3^ cells we saw that the D492^SPRY2-NS^ cells showed normal expression of SPRY2 at the lobular tips whereas the D492^SPRY2-KD3^ cells showed markedly suppressed expression (Fig. 5F).

### Endothelial cells stimulate EMT in D492^SPRY2-KD^ cells

**Figure 6:**
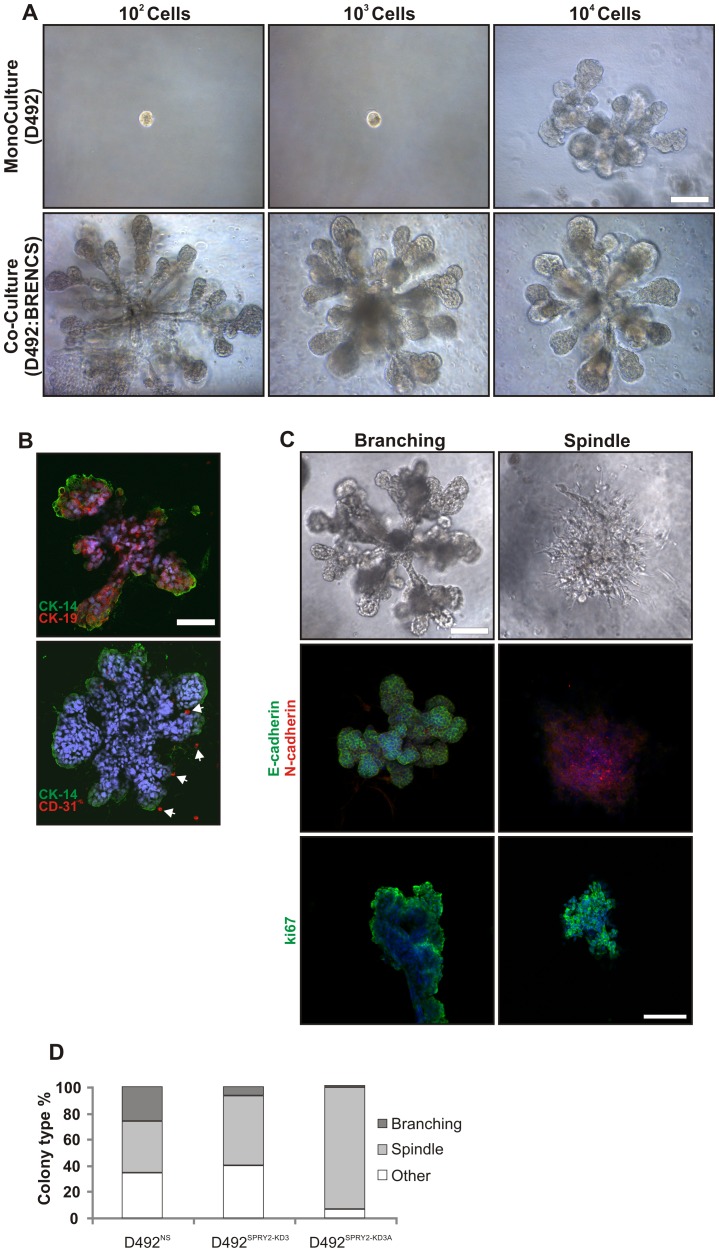
Epithelial integrity is disturbed in SPRY2 KD cells when co-cultured with endothelial cells. *A) Endothelial cells stimulate growth of D492 cells.* When plated in 3D rBM culture with breast endothelial cells (BRENCs), D492 cells can form complex branching colonies from as little as 100–1000 cells compared to 7×10^3^ –10^4^ in 3D monoculture. *B) D492-derived branching structures form bi-layered epithelium with BRENCs positioned extralobular.* The branching colonies are bi-layered and polarized structures as evidenced by the expression of the myoepithelial marker CK14 on the outer side and the luminal epithelial marker CK19 on the inner side (upper figure). In co-cultures endothelial cells stay as single cells positioned outside the branching structures as seen with CD31 staining (lower figure, arrows). Bar  =  100 µm. Sections counterstained with TOPRO-3 nuclear stain. *C) Phenotypes of D492 in co-culture with BRENCs.* In co-culture with endothelial cells D492 cells form both branching- and spindle-like colonies. The branching colonies show strong expression of E-cadherin, while spindle like colonies have undergone EMT as evidenced by cadherin switch from E- to N-cadherin. Staining for the proliferation marker ki67 shows that both the branching and spindle like colonies are viable and growing. Bar  =  100 µm. Sections counterstained with TOPRO-3 nuclear stain. *D) Spry2-KD cells show an increase in the spindle-like morphology.* While D492^NS^ cells form about 40% spindle-like colonies there is a significant increase in the D492^Spry2-KD3^ cells up to 65%. The D492^Spry2-KD3A^ form almost exclusively spindle-like colonies in co-culture with endothelial cells.

We have recently shown that endothelial cells support morphogenesis and improve clonal efficiency in both lung and breast epithelial cells [35], [36]. Furthermore, we have shown that breast endothelial cells (BRENCs) induce EMT in D492 cells [37]. Interestingly, when D492 cells are co-cultured with BRENCs we also see marked stimulation in branching morphogenesis at clonal dilution (Fig. 6A). Branching TDLU-like structures in co-culture were generated from as few as 100 D492 cells in 300 µl rBM compared to the usual 10,000 cells used in monocultures in this assay (Fig. 6A). Immunophenotypic characterization of the TDLU-like structures generated in co-cultures revealed distinct luminal- and myoepithelial differentiation as shown by expression of cytokeratin 19 and 14, respectively (Fig. 6B). Dual immunostaining against cytokeratin 14 and CD31 demonstrates the perilobular location of endothelial cells surrounding the TDLU structures (Fig. 6B). Thus, TDLU-like colonies generated in co-cultures with BRENCs mimic TDLÚs *in situ* with a bi-layered epithelium consisting of an inner layer of luminal epithelial cells, an outer layer of myoepithelial cells and an extralobular location of endothelial cells.

To see if SPRY2 KD cells responded differently to BRENCs we set up co-cultures with D492^SPRY2-NS^, D492^SPRY2-KD3^ and D492^SPRY2-KD3A^ cells. D492^SPRY2-NS^ formed 50% spindle-like (EMT) colonies and 40% branching colonies while D492^SPRY2-KD3^ cells formed over 65% spindle-like colonies (Fig 6C and D). The D492^SPRY2-KD3A^ clone which produced larger and a higher number of branching colonies in the monoculture was also used in the co-culture and interestingly they exclusively produced spindle-like colonies. The spindle-like colonies were similar to previously reported endothelial-induced EMT colonies [37] and this was confirmed by an E- to N-cadherin switch (Fig. 6C). The proliferation was similar in both branching and spindle colonies as evidenced by Ki67 expression. Partial EMT is known to occur during branching and this suggests that SPRY2 might regulate branching through temporal suppression of EMT during the branching process possibly through inhibition of RAS/ERK pathway. SPRY2 knockdown cells might thus be prone to both increased branching and more susceptible to full EMT with formation of spindle-like EMT colonies under co-culture conditions.

## Discussion

The sprouty protein family is increasingly recognized as a key regulator of receptor tyrosine kinase signaling in different species and organs where SPRY2 has captured most attention [43]–[45]. Furthermore, SPRY2 has been shown to be downregulated in a number of cancers, including breast cancer [29], [30], [46]. In this study, we have analyzed the expression of SPRY2 in the mouse and human mammary gland.

In the human breast gland SPRY2 was equally expressed in ducts and TDLU, and its expression was most prominent within luminal epithelial cells. We also demonstrate that the expression of total SPRY2 is low in virgin mouse mammary glands but is focally increased at branching tips during pregnancy and reaches maximum expression during lactation. The presence of phosphorylated SPRY2 was most profound during pregnancy where it seems to dampen signaling through RAS/MAPK pathway. In support of this the pERK1/2 levels increase during lactation when a reduction is seen in pSPRY2. Previously, Lo et al. [29] demonstrated by *in situ* hybridization that SPRY2 was highly expressed in pregnant mouse mammary glands but decreased during the lactating stage. In our study total SPRY2 expression was high at lactating stage but pSPRY2 level was low.

The functional role of sprouty in branching morphogenesis during trachea development in Drosophila was first demonstrated in spry-/- mutants which showed excessive branching [47]. Similarly, Tefft et al. [48] demonstrated that inhibition of SPRY2 expression in mouse embryos at E11.5 produced a significant increase in lung branching. Development of the uretic bud is another example of controlled branching morphogenesis that is regulated by sprouty proteins [43]. In the nephric duct, cells with high Ret tyrosine kinase receptor expression preferentially move to the dorsal nephric duct adjacent to the metanephric mesenchyme where they form the first uretic bud. Interestingly, SPRY1-/- mutants show elevated expression of RET and increased branching [49]. These data collectively demonstrate the regulatory role of sprouty proteins during branching morphogenesis in various epithelial organs.

Mouse studies have shown that the mammary organoid branches and migrates by bifurcation and collective migration [50]. Furthermore, end bud and TDLU formation requires growth factor induced cell proliferation and studies show that this cell proliferation is mediated through ERK1/2 [7], [50]. These results are consistent with our data. D492 cells in 3D culture capture, by collective migration, the morphogenic process in the mammary gland, including the formation of TDLU like structures. SPRY2 expression was most prominent at the peripheral branching buds whereas it was significantly reduced in clefts/furrows both in *in vitro* 3D cultures of human breast epithelium and in the growing mammary gland in pregnant mice. The same expression pattern was also seen for p-EGFR. This indicates that the D492 cell line activates both EGFR and SPRY2 pathways during branching morphogenesis.

It is becoming clear that the stromal microenvironment plays a critical role in tissue morphogenesis of many organs, including the breast [51]. It is also widely acknowledged that cancer progression is dependent on signals from the surrounding microenvironment [52]. Fibroblasts and extracellular matrix molecules, such as laminin, fibronectin and extracellular matrix-entrapped growth factors have received much attention [28]. Our recent results demonstrate that endothelial cells stimulate growth and morphogenesis of breast epithelial cells [35] and induce EMT [37]. We have also shown that endothelial cells can induce bronchial epithelial cells with stem cell properties to generate bronchioalveolar branching structures in 3D culture [36]. Interestingly, in co-culture of endothelial cells and D492 we see a dramatic increase in TDLU formation. This further demonstrates the proliferative and morphogenetic induction potential of endothelial cells.

During branching morphogenesis epithelial cells need to transiently activate critical mesenchymal properties to be able to invade the surrounding matrix. This mesenchymal transition proceeds gradually under tight control of morphogenetic signals and under regulation of the microenvironment [53]. Branching morphogenesis can therefore be regarded as partial EMT that is under tight control from the surrounding microenvironment or from within the invading cell. When we suppress SPRY2 expression in D492 we see hyperplasia-like effects and increased branching morphogenesis. Furthermore, when the SPRY2 knockdown cells are co-cultured with endothelial cells they showed increased EMT susceptibility. We have previously shown that endothelial-induced EMT in D492 is partially mediated by hepatocyte growth factor (HGF) [37]. The increase in EMT after SPRY2 knockdown suggests that SPRY2 might be critical in temporally regulating EMT during branching morphogenesis through modulation of RTK signaling.

## Conclusion

Our data suggest that breast epithelial branching morphogenesis is regulated by SPRY2. Furthermore, our data indicate that SPRY2 is an important regulator of epithelial integrity as SPRY2 knockdown cells are prone to endothelial induced EMT.

## Supporting Information

Figure S1 **Expression of SPRY2 in virgin, pregnant and lactating gland.** SPRY2 is present at all developmental stages in the adult mammary gland with highest expression seen during lactation. Actin used as a loading control.
(TIF)

Figure S2 **SPRY2 knock down result in increased pERK activity.** Knock down of SPRY2 result in approximately 20% increase in pERK activity. Actin used as a loading control.
(TIF)
