# Supplementary figures and images for: Expression and Functional Role of Sprouty-2 in Breast Morphogenesis
Source: PLoS One. 2013 Apr 3;8(4):e60798. doi: 10.1371/journal.pone.0060798 (PMC3616012; doi:10.1371/journal.pone.0060798)

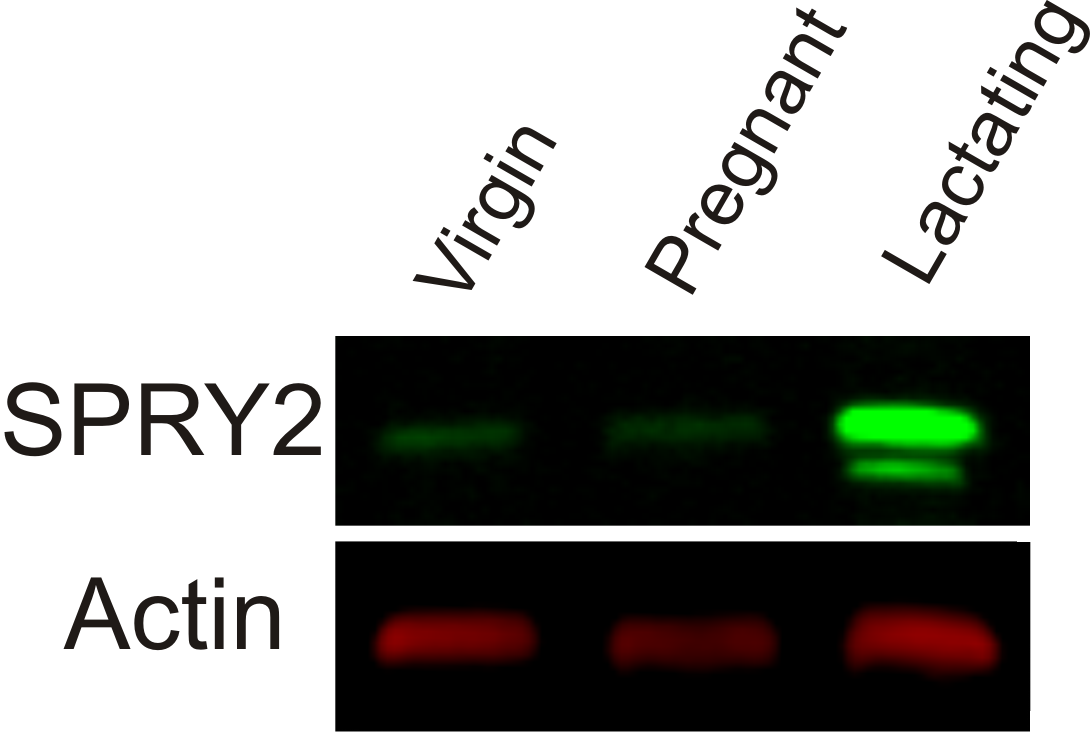

Supplement: Figure S1 — Expression of SPRY2 in virgin, pregnant and lactating gland. SPRY2 is present at all developmental stages in the adult mammary gland with highest expression seen during lactation. Actin used as a loading control. (TIF) [file pone.0060798.s001.tif]

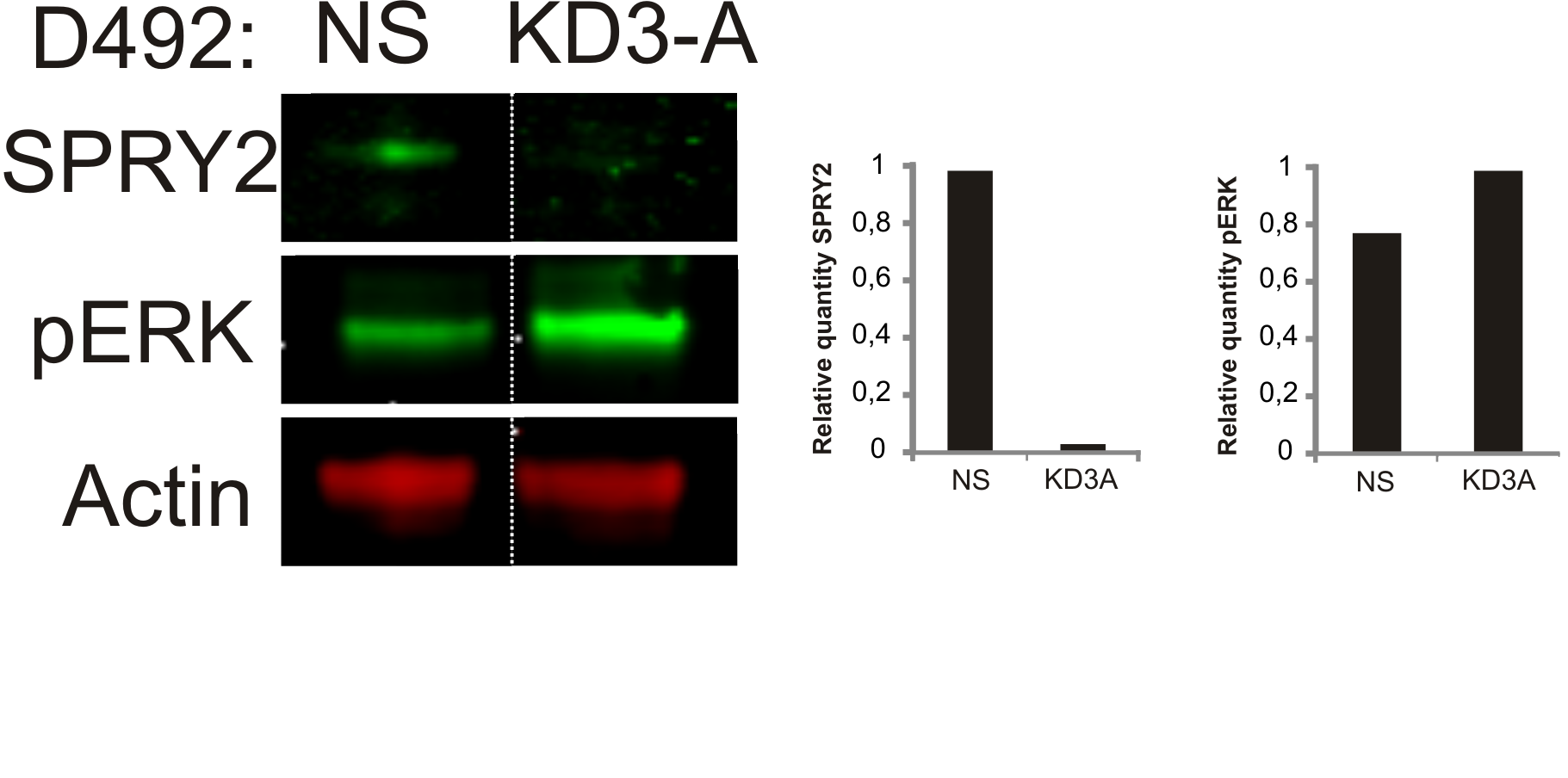

Supplement: Figure S2 — SPRY2 knock down result in increased pERK activity. Knock down of SPRY2 result in approximately 20% increase in pERK activity. Actin used as a loading control. (TIF) [file pone.0060798.s002.tif]
